# An Unusual Annular Contraction of the Uterus during Labor

**Published:** 1875-10

**Authors:** E. Mason

**Affiliations:** Wetumpka, Alabama


### AN UNUSUAL ANNULAR CONTRACTION OF THE UTERUS DURING LABOR.

By E. MASON, M.D., of Wetumpka, Ala.

On July the 27th, 1875, 5 o'clock P.M., I was called to see Mrs D. J. S., a short, stout-built lady, of lymphatic temperament, in her first labor. I was informed that she had been in hard labor since 1 o'clock, at which time the membranes were ruptured and a very large quantity of water discharged. Her general condition was good, pulse normal and skin very pleasant. The character of the pains induced me to think that she was in the last stage of labor, and I made an immediate examination. I was astonished to find the os uteri dilated scarcely enough to admit my index finger; it was, however, very soft and dilatable, but quite thick. I could reach the presenting part of the fœtus with some difficulty, which I found to be the breech. Although the pains seemed to be violent expulsive efforts, returning about every five minutes, yet there was no downward pressure. The uterus seemed to be morbidly exercised; there was no power in the fibres which tend to force the fundus towards the os, while those that approximate the sides of the womb seemed to hold the fœtus perfectly still. I gave a large dose of castor oil, which acted well by 8 o'clock, and I then gave about a half grain of sulphate of morphine. Her pains soon became less in frequency and force, and she slept a good deal during the night.

At 3 o'clock on the morning of the 28th her pains had become severe again, without any change in their character. I made another examination, and was still more surprised to find the os but very little more dilated and the presenting part just where I had left it at my last examination. I now watched the pains very closely, and found them very peculiar. During the first part there would be slight downward pressure, then sudden spasmodic pressure upwards. The position at the womb was very much to the right side, which was only partially remedied by keeping the patient on her left side. About 10 o'clock she complained of some pain in her head, and her pulse had become too full. I bled her and gave another dose of morphine, hoping to relax the system and overcome this novel condition she was in. After several hours of comparative rest, the pains assumed their original character, without accomplishing anything effective towards delivery. I had already announced to the family the great hazard to which the child was exposed.

In the afternoon it was very clear that my patient was becoming exhausted, and that she would not be delivered by the efforts of nature alone. About this time my attention was directed to the appearance of the patient's abdomen, which had caused her lady friends to predict that she would have twins. The uterus slightly represented two tumors, one above and the other below, which peculiarity could be recognized by careful examination with the hand. My friend Dr. Green, whom I had sent for several hours before, arrived about 6 o'clock P.M., and we proceeded to attempt a delivery. The os was still undilated, and the uterus hung down over the presenting part like a bag. We could dilate the os sufficiently to pass the forceps, but the breech, which presented towards the right acetabulum, was so high that it was impossible to apply the instrument. We made many futile attempts to use the blunt hook and our hands, to bring down the breech. Failing in all these efforts, I determined to pass my hand into the uterus itself, and, if, possible, find out the difficulty. Having oiled my hand well, after long continued pressure I succeeded in passing it over the right hip of the fœtus until I could reach the groin with my finger; my hand was now even with the umbilicus of the patient. By careful examination when there was no pain, I found the body of the child embraced by a band-like contraction, which became hard and tense during pain. Finding that I did not have power in my hand to overcome this contraction and bring the breech down, I passed a blunt hook over the right thigh into the groin, and while Dr. Green kept up gentle traction, I manipulated with my hand. After we succeeded in getting the shoulders to pass the contraction, we had no further trouble. As soon as the child was delivered, I passed my hand again into the uterus to remove the placenta, and found it attached to the right side, just above the contraction; I removed it without much difficulty. I applied cold externally and gave a dose of Squibb's fluid extract of ergot, and very soon had a firm contraction of the whole organ. Hemorrhage was very profuse for a few minutes. The infant was dead, but the mother recovered without an unpleasant symptom.

I have been thus minute, and perhaps tedious, in reporting this case, which may not be as interesting to others as it has been to me. It is something new to me; I have never seen nor read of a case simulating "hour-glass contraction" of the uterus before the expulsion of the fœtus. I did not give the patient chloroform because her friends were very much opposed to her taking any anæsthetic.

